# The views of migrant health workers living in Austria and Belgium on return migration to sub-Saharan Africa

**DOI:** 10.1186/s12960-016-0129-4

**Published:** 2016-06-30

**Authors:** Annelien Poppe, Silvia Wojczewski, Katherine Taylor, Ruth Kutalek, Wim Peersman

**Affiliations:** Department of Family Medicine and Primary Health Care, Ghent University, Ghent, Belgium; Unit Ethnomedicine & International Health, Department of General Practice & Family Medicine, Medical University of Vienna, Vienna, Austria; Department of Primary Care Health Sciences, Oxford University, Oxford, UK

**Keywords:** Circular migration, Healthcare, Health workers, Human resources, Return migration, Sub-Saharan Africa

## Abstract

**Background:**

The negative consequences of the brain drain of sub-Saharan African health workers for source countries are well documented and include understaffed facilities, decreased standards of care and higher workloads. However, studies suggest that, if migrated health workers eventually return to their home countries, this may lead to beneficial effects following the transfer of their acquired skills and knowledge (brain gain). The present study aims to explore the factors influencing the intentions for return migration of sub-Saharan African health workers who emigrated to Austria and Belgium, and gain further insight into the potential of circular migration.

**Methods:**

Semi-structured interviews with 27 sub-Saharan African health workers in Belgium and Austria were conducted.

**Results:**

As mentioned by the respondents, the main barriers for returning were family, structural crises in the source country, and insecurity. These barriers overrule the perceived drivers, which were nearly all pull factors and emotion driven. Despite the fact that only a minority plans to return permanently, many wish to return regularly to work in the healthcare sector or to contribute to the development of their source country.

**Conclusion:**

As long as safety and structural stability cannot be guaranteed in source countries, the number of return migrants is likely to remain low. National governments and regional organizations could play a role in facilitating the engagement of migrant health workers in the development of the healthcare system in source countries.

## Background

Annually, thousands of skilled workers emigrate from sub-Saharan Africa, of which a considerable number are health workers [1]. The reasons for their migration are diverse, and include lack of opportunities for professional development, unavailability of working equipment and supplies, heavy workloads, low wages, and the threat of political instability and violent conflict in their home countries (push factors), as well as better remuneration and working conditions, educational opportunities, and family reunification in the host countries (pull factors) [2–12]. The negative implications of such brain drain for the healthcare systems in source countries are well documented, and include the understaffing of medical facilities, which leads to a decrease in the standard of care as well as to high workloads, and the lack of treatment for some diseases [13, 14]. Additionally, as estimated by Mills et al. [15], the emigration of sub-Saharan African health workers implies a considerable loss of investments in medical education.

Nevertheless, despite the reported losses for source countries, studies indicate that beneficial effects may also ensue, including financial remittances [16–19], opportunities to develop professional networks with colleagues in high-income countries [13], and the potential of return migration with increased skills [20]. According to some economists, high rates of return migration of skilled workers after temporary stays abroad can re-supply the highly educated population in source countries and thus increase countries’ average productivity, especially if health workers return after gaining experience and skills in a more advanced economy [21]. While working in a high-income country, a health worker may form a professional network and develop skills and expertise that can be applied when returning home [22–24]. A successful return and reintegration contributes to the reinforcement of financial, human and social capital in the source country [25]. As Asampong et al. [26] confirm, returning health professionals contribute to the development of the health sector through the transfer of acquired skills and knowledge. Some observers believe that rates of return are high enough to use the terms ‘brain circulation’ or ‘professional transience’ instead of ‘brain drain’ to describe the global migration of skilled professionals [21]. However, for sub-Saharan African health workers specifically, the number of migrated health workers who return home at any point appears to be relatively small [12, 14, 17]. Notwithstanding the rather infrequent occurrence of return migration among migrated sub-Saharan African health workers, the phenomenon of return migration and circular migration has received growing attention over the last two decades as it is considered an important instrument to partially reverse the negative effects of the large scale professional emigration of health workers [27].

Previous research has illustrated that the migration decision is a complex process, influenced by several factors, and that health workers’ motivations to stay or return home change over time [17, 28, 29]. Different types of migrants can be identified, including livelihood, career-oriented, backpacker, commuter, undocumented, returned, family, and safety and security migrants; findings indicate that there is some fluidity between these categories due to the changing motivations of migrant health workers [28].

From the perspective of return migration, stay factors – those that keep migrants in the destination country, including reluctance to disrupt family life, children’s education, career paths, lack of incentives to return home, and a higher standard of living in the recipient country – are important [12, 26]. Asampong et al. [26] indicated that the reasons for return migration are fairly similar to those for initial migration, but that pull factors (desire to share knowledge and skills, complete a project, reunite with family, etc.) dominate the return, whereas push factors were more influential in the initial migration. Taylor et al. [30] recently studied the ongoing ties and future plans of South African health workers who migrated to the United Kingdom, and found that the main barrier to returning home was usually the development of stronger family ties in the United Kingdom compared to in South Africa. Additionally, factors that initiated the original migration decision, such as security and education, remained important to stay in the United Kingdom. Nevertheless, the migrated health workers maintained strong links with South Africa through family, friends and professional links and the majority still saw South Africa as their ‘home’ and wished to repay something to their community.

Despite the growing attention on return migration, only a few studies have investigated whether migrated sub-Saharan African health workers do return to the source country, the factors involved in their decision-making and the actual contribution of the returnees to the local healthcare system [30, 31]. There have been studies on return migration of health workers, but mainly in countries actively recruiting health workers from overseas, such as the United Kingdom or Ireland [30]. Other studies have focused on the intentions to stay, aiming to use the results to develop interventions which might influence migrants’ decisions to remain in the destination country, and focusing chiefly on nurses originating from countries other than those within sub-Saharan Africa and who have recently migrated [32, 33].

Contrary to the United Kingdom or Ireland, most countries of the European Economic Area do not actively recruit health workers trained outside it and, consequently, they do not receive such large-scale migration of health workers [34]. Nevertheless, despite the overall number of migrating health workers to these countries being small, the impact on the medical workforce for the source countries is significant [34].

Given that the context and aim of previous research is somewhat different and that the results of migration studies are specific to both source and destination countries [29], the aim of the present study was to explore the factors influencing the intentions for return migration of emigrated sub-Saharan African health workers in Austria and Belgium, two countries that do not actively recruit foreign-educated health workers, and to learn more about the potential of circular migration and the future professional plans upon return.

## Case presentation

### Methods

#### Design

This study presents data collected through semi-structured interviews with migrated sub-Saharan African health workers in Belgium and Austria [7, 11]. Data collection took place within the framework of the HURAPRIM project (EU-FP7) on human resources in primary healthcare in sub-Saharan Africa (http://www.huraprim.ugent.be/). Participants had to be born in sub-Saharan Africa, be professionally trained in sub-Saharan Africa in medicine (medical doctors and medical assistants) or nursing, and be living in Belgium or Austria at the time of the study.

In order to identify potential participants, we used a variety of recruitment strategies. Firstly, several organisations, such as African migrant organisations, organisations which undertake the counselling of newly arrived migrants and language centres, were contacted with a request to identify potential participants. In Austria, contacts were also made with the Austrian Medical Chamber. Organisations that could not provide names or addresses for privacy reasons distributed a flyer explaining the study to their members/clients so that they could contact us themselves. Secondly, we contacted nursing homes and hospitals with a request to spread the flyers and identify potential participants among their personnel. Thirdly, the call for participants was circulated on different online fora and the flyer was distributed in neighbourhoods with a high concentration of African migrants. Finally, a snowballing technique and informal networks were used to identify potential participants. Recruitment through African migrant organizations yielded few participants and most participants were recruited through the snowballing technique (mainly through former respondents). We aimed to compile a heterogeneous sample, based on age, source country, training, and length of time resident in the destination country.

#### Data collection

The Department of Family Medicine and Primary Health Care (Ghent University) was responsible for the data collection in Belgium. The Department of General Practice and Family Medicine, Ethnomedicine and International Health Unit (Medical University of Vienna) collected data in Austria.

In both countries, an experienced female researcher conducted the interviews. The data collection procedures were similar. Interviews took place in English, Dutch, French or German, at a place chosen by the interviewee: the home of the participants, their work place or at the researcher’s office, and lasted 30–90 min. Participants received an information letter before the interview about the practical aspects and goals of the study. Written informed consent to participation was obtained for all participants. A semi-structured topic list was used as an interview guide. The determination of the main topics was preceded by a review of the literature on the migration of sub-Saharan African health workers to high-income countries [35]. The topics included their intentions for return migration, the migration experience, changes needed in the source country in order to retain health workers, experiences in the destination country, and transnational ties [7, 11, 36]. The interviews were recorded and transcribed verbatim by the interviewer or another member of the research team. Interviews were conducted until thematic saturation was reached or, in the case of Austria, until no further participants could be found using the methods described. The research team completed 27 semi-structured interviews with migrant health workers, of which 17 were in Belgium and 10 in Austria. Interviews were conducted between October 2011 and April 2012.

### Data analysis

Data was coded using an open coding process. In an iterative process, a label or code was applied to selected text, and codes were clustered, compared and sorted into distinct and comprehensive themes and sub-themes. Themes were compared and sorted in a process of constant comparison [37] in order to refine existing codes and identify new ones [38]. The coding and developing of themes was done by AP, using NVivo 10 software. A senior researcher (WP) read through all the transcripts and also independently coded the first interviews. After several iterations and discussion with the co-authors who had conducted or were familiar with the content of the interviews, the results were finalised and are presented below.

#### Ethics statement

The Ethics Committee of Ghent University Hospital approved the Belgian study (Ref.: 2011/552). The Austrian study was approved by the Ethics Committee of the Medical University of Vienna (EK-Nr: 989/2011).

### Results

Twenty-seven participants were interviewed (12 men and 15 women). The youngest participant was 26 years old and the oldest 59. Overall, 18 participants were trained as medical doctors in the source country, seven as nurses, and two as medical assistants. The participants originated from 16 different countries in sub-Saharan Africa (Table 1) and all lived in Belgium or Austria at the time of the interview. The time since their original migration from the source country ranged between 2 months and 34 years. The most important reasons for the initial migration to Belgium and Austria (both countries without a history of active recruitment) were educational purposes, political instability or insecurity in the source country, and family reunification [7]. Most of the respondents were not working in the same profession as in the source country; some were working in the healthcare sector but in a role for which they were overqualified, others were working outside the healthcare sector, and others were not working at all. This was mainly due to problems with bureaucracy and legal issues. Many were also involved in procedures of diploma equivalence, or had already started the necessary extra years of study to reach equivalence.

**Table 1:** Cohort characteristics

	*n* = 27
Sex	
Male	12
Female	15
Age, years	
20–30	3
31–40	14
41–50	5
50+	5
Time since leaving source country, years	
< 5	8
5–10	7
> 10	12
Professional background in the source country	
Medical doctor	18
Nurse	7
Medical assistant	2
Source country	
Congo	5
Rwanda	4
South Africa	4
Guinea	2
Nigeria	2
Angola	1
Burundi	1
Gabon	1
Ghana	1
Ivory Coast	1
Senegal	1
Somalia	1
Sudan	1
Tanzania	1
Uganda	1

A variety of factors influencing the motivation to return were identified following the interviews (Table 2).

**Table 2:** Factors involved in the intentions to return

Barriers	Drivers
• Family ties • Institutional crisis in source country • War and internal conflicts in source country	• Family • Emotion-driven factors: - Fact that it is their home country - More satisfactory to work in source country - Feeling of responsibility

#### Barriers for return migration

An important influential factor for original migration was having family in their European destination country; nevertheless, since most of the respondents arrived in the destination country childless, this implied that they could move rather freely. However, once migrating health workers had settled and had children, their freedom of movement became limited, with some of the participants reporting that their first concern was their children’s stability and wellbeing. Though some of the participants would personally like to return, they chose their family’s stability as a priority. Some participants stated that, until their children became of age to decide about their future, they did not want to change the educational and social environment their families were are accustomed to.

“*But now I have children who are going to school and I don’t intend to move them away from their school. It’s too different, to just move them during their education.*” (Belgium 6, Medical doctor)

“*But for my husband I don’t know, and for my children who were born here it would maybe be difficult with the school.*” (Austria 5, Medical doctor)

Second, institutional crises in their source countries, in combination with the positive living conditions in Europe, played a role in their lack of intention to return. Further, staying in Europe was also perceived as beneficial for their children in this context.

“*Now there is a huge structural crisis in* [source country]*. Education, healthcare, there are many problems. So I don’t think it is a good moment to return now.*” (Belgium 8, Medical doctor)

“*It will take many years before* [source country] *finds its place. So, I don’t want my children to grow up in such living conditions.*” (Belgium 10, Nurse)

“*We also benefit from a better education, and a better healthcare here.*” (Belgium 16, Medical doctor)

Third, war and internal conflicts were, in some cases, the reason for the initial migration, and continued to represent a barrier to return migration. The situation in Europe is stable and safe, which is not the case in some of the source countries.

“*At this moment, all I want is to return to my country. The only thing I have here is my life. It’s to protect my life, and that’s what I consider priority now. Because they have killed many people from my ethnic group in* [source country]*, many.*” (Belgium 7, Medical doctor)

One participant indicated that war implies safety risks as well as instability, which makes it hard to find a job and develop a career.

“*But mainly for me, the key element, I was looking at my career. I was saying: Okay, even if I turn back now, there is war. My career will end, just like that.* […] *Always I’m saying, it’s useless, it’s lost to send someone in a place where he will be forgotten, where he will do nothing. Better to stay in another place and at least to talk, at least go in a meeting and say: this is the situation and this could change. But if you are lost there, they have lost you forever.*” (Belgium 6, Medical doctor)

The abovementioned factors were the most reported reasons for staying in Europe. Nevertheless, there were other, more personal factors that were mentioned to a lesser extent, including health reasons, financial issues, working conditions in the source country, and no possibility for legal return migration. Respondents from South Africa also cited criminality as an important reason not to return.

“*Criminality is a huge problem in SA. It has been for a long time, but I always said, well, it’s just statistics. But after a while, everybody I knew had become a victim, us too. We had a carjacking and of course that’s not a pleasant experience. And I won’t say that it is the reason why we left, but it definitely is part of the reason why we won’t be going back.*” (Belgium 4, Medical doctor)

#### Drivers for future return migration

Despite family being the most commonly mentioned barrier for return migration, for those whose extended family was still living in the source country, this represented a driver for return migration.

“*It’s better to return, to be with our family.*” (Belgium 16, Medical doctor)

Secondly, the source country was still their home country, the place where they were raised and feel at home. Thus, the desire to inculcate their African identity to their children represented a driver for return migration.

“*So, in my opinion, of course it’s very nice to go back to your home country, and I’d like that.*” (Belgium 6, Medical doctor)

“*I want my children to also know Africa.* […] *They say that you are either white nor black. It means that we try to give the African culture to our children, but we cannot because the environment here is not the same. And we can’t seem to integrate fully here neither, because there is always the influence of our family. I would love my children to have more African roots. That they know the values of Africa, from home.*” (Belgium 16, Medical doctor)

Additionally, many believed that working in the source country is ultimately more satisfactory due to the influential difference they could exert.

“*I know they need my help best in Africa than here, when it comes to delivery of medical help, services. People in Africa may need me more. Because there are few doctors and many patients, unlike here, we have many doctors, sometimes few patients. So if you look at delivery of services, then I may tell you that Africa, when it comes to medicine they need me.*” (Belgium 1, Medical doctor)

“*After studying I thought I could be more helpful there* [source country]*. It was extremely satisfying to work as a doctor there because you feel you can really do something there.*” (Austria 3, Medical doctor)

Feelings of obligation towards the source country which offered them the opportunity to study as well as of the need to contribute something in return were also reported as drivers.

“*Well, I studied there for six years, I got a scholarship for those six years from the* [source country] *state. Not only that but I just feel like, as a* [nationality]*, I am obliged to do something for the country.*” (Austria 7, Medical doctor)

“*One day I would like to return to help the population of my country. To see what we can do, contribute.*” (Belgium 13, Medical doctor)

A small minority of the respondents mentioned discontent with life in Europe due to financial issues, personal struggle and a feeling of not belonging, as a reason for return migration.

“*Many people I know have returned to* [source country]*. They experienced that life over here is too expensive. What are they doing here? They suffer because of all the payments and bills. Everybody returns.*” (Belgium 12, Nurse)

“*I could work perfectly in Africa. If you give me the chance to return, I fly away immediately. What are we doing here? With all the harassments? We could perfectly live there, with two doctors we would earn enough money, we could do a lot. I tell you what is in my heart. My heart is in Africa.*” (Belgium 9, Medical assistant)

#### Prospects for return

A number of respondents saw Europe only as a temporary destination and their ultimate goal was to return. Initial migration was due to further education or to save money in order to use it upon their return home; return migration was planned as soon as their objectives were achieved in Europe.

“*I still want to practice as a medical doctor, definitely. My wife and I are planning to go back to* [source country] *to have a hospital, to manage a hospital. Also with my management background here in Austria, able to try and run a hospital in* [source country]*. But it also needs funds I mean, you have to get money to have this place built and all that and all that. So, this is what we are planning on doing, this is basically my big future.*” (Austria 1, Medical doctor)

“*I want to go back home one day to establish myself in medicine. So I’m working now, with my people also in the UK. We are coordinating. But I am doing some cash flow work here now. Because I wanted to practice my medicine one day, so for now I’m just hovering, doing one or two things. Later, when I have everything I need, then I go back to Africa.*” (Belgium 1, Medical doctor)

“*But from my side, I think I will return, yes. When I have completed my study. Even my husband, he doesn’t want to stay here. It’s in our search for a better life. But when you have a minimum, it is better, we are better back home, we can help better over there.*” (Belgium 16, Medical doctor)

Nevertheless, despite their absolute intention to return, they were also aware that some factors may be outside their control or may interfere over time.

“*It also depends on how long it will take to get diploma equivalence here, how many additional years of study I’ll have to do. And I prefer to do a specialisation as well.*” (Belgium 8, Medical doctor)

Not all those with a desire to return wished to fulfil a professional role in healthcare in their home country. Some stated that they would consider returning when their professional career was over in order to enjoy retirement in the country they still consider home.

“*I do think about it, I actually think I’m going to return back home, not necessarily to go and work back there but definitely I want to return back home.*” (Austria 6, Medical doctor)

#### Circular migration and temporary return migration

Several participants reported maintaining professional contacts in their source country and regularly returning to work in the healthcare sector. Indeed, circular migration for professional reasons fosters the creation of professional networks, the maintenance of skills, and the establishment of opportunities for an eventual permanent return migration.

“*I return once a year. To visit, but also to work in the hospital. Because I don’t want to lose my experience. I want my skills to stay sharp, my* [source country] *skills. Because I have to stay able to perform as a general physician. I return for one month, and within this month, I spend three weeks in the hospital. I renew my inscription as a doctor each year, in* [source country].” (Belgium 15, Medical doctor)

“*And my feeling is always to keep links with those countries. That’s why I have so many projects in Africa. I’m always looking.*” (Belgium 6, Medical doctor)

For others, circular migration occurs due to an underlying emotional attachment and sense of responsibility towards the source country, without having the intention to return permanently. These respondents believe that their skills and knowledge are needed in their source country and are therefore eager to help, doing so by returning for short time periods and providing assistance in healthcare services, thus contributing to the development and reinforcement of healthcare services in the source country.

“*I work privately and with associations in* [source country]*, for example we have two projects where we work with schools.* […] *We built a library that is free for everyone but especially for school children.* […] *For six years I got a scholarship from the government, and it is not only that as a* [nationality] *I feel I have to do something for my country.*” (Austria 7, Medical doctor)

“*I have plans to go to* [source country] *to do some work there, especially those areas which are peaceful, and I am trying with some friends, Austrian friends, we are trying to do that but I don’t know when it will be, but we are trying to, to do something in* [source country].” (Austria 2, Medical doctor)

“*I think if there is a need, and if there is change in the political situation in my country, then why not, I will return back to make a difference, to do something that I know that it is very important. I can imagine that I am back there and doing what I am doing here also there.*” (Austria 4, Medical doctor)

“*Return? When I’m retired, maybe even before. Here in my city there is a group of people, all retired, they want to go back on missions and contribute there as a volunteer.*” (Belgium 17, Nurse)

### Discussion

This paper is among the first to explore intentions for return migration among sub-Saharan African health workers resident in European countries that do not actively recruit, focusing on how such return migration would be beneficial for the source countries. The above analysis revealed that family ties, structural crises in the source countries, and the better living conditions in Europe, as well as insecurity resulting from war and conflict in source countries, were all factors encouraging health workers to remain in Europe. Nevertheless, respondents also indicated that reunification with their extended family, a desire to live in their home country, the belief that working in sub-Saharan Africa is ultimately more satisfying, and a sense of responsibility towards their source country leading them to consider it their duty to contribute to its healthcare system, were all reasons for return migration. Discontent with life in Europe, criminality in the source country (specifically for South Africa), health reasons, working conditions, and no possibility for legal return, were all also mentioned as both drivers and deterrents for return migration, albeit to a lesser extent.

In accordance with the findings of Asampong et al. [26], the main drivers for return migration are all pull factors. Rational factors seem to dominate the balance in favour of remaining in Europe, while the reasons cited for returning are more emotionally driven and situated at the micro level. Despite at least one of the pull factors being mentioned by nearly all respondents, there were other factors, mainly at the meso and macro levels, overruling these. Many testified that contextual, structural and situational factors are important influences. Those who originally migrated because of insecurity would not consider returning for as long as conditions in the source country remain unchanged. Additionally, social conditions, the political climate and criminality are important situational barriers to return migration, even for those who did not cite these as their original reason for migration. Thus, as long as there is no change in the social, political and safety conditions in source countries, the number of health workers permanently returning will remain rather small.

Some health workers described a definite intention to return and referred to their time in Europe as temporary. However, they were aware that, over time, their opinion could change due to factors not previously considered (having children, longer than expected time required to get diploma equivalence, further study, etc.). This was also mentioned by those who had been living in Europe for a longer time. Indirectly, time plays an important role; the longer migrants stay, the harder it is for them to return, as indicated by Humphries et al. [28] and Akl et al. [29].

Only a minority of the respondents intended to return permanently; of these, some intended to return as health workers, whereas others planned to work in different areas or return following retirement. This finding supports the statement of the World Health Organisation indicating that the number of migrated health workers who eventually return home is relatively small [14].

The varied responses obtained during the interviews highlight the large spectrum between permanent migration and definite return migration. As Dodani and LaPorte [39] indicate, the increasing opportunities for communication, travel and collaboration between low- and high-income countries imply that brain gain/circulation can be established through methods other than through a permanent return. Despite the fact that only a minority of our respondents wished to permanently return to work in healthcare, the interviews highlight the potential for brain circulation based on temporary visits. Constant and Zimmermann [40] defined circular migration as “*the systematic and regular movement of migrants between their homelands and foreign countries typically seeking work*”. These movements may precede a permanent return, but our interviews show that this is certainly not the case for everyone. The majority of the respondents testified that they view circular migration as a means to remain linked with their source country and to contribute to the development and strengthening of its healthcare sector without the necessary prospect of permanent return. For those who wish to return but are confronted with unsurmountable barriers, circular migration appears to be a suitable alternative.

Our interviews highlight the migrated health workers’ willingness to temporarily return to their source country to be actively involved in the development of its healthcare system, which is in line with the conclusions of Stuart and Russell [41], namely that the diaspora is now moving from a financial support role through remittances to a more active role in strengthening the social and economic development of their source countries. However, as indicated by several of the respondents herein, although they are eager and willing to contribute, they do not really know how to realize this aim.

Nevertheless, history shows several examples of regulated circular migration based on agreements between the involved countries [27, 42]. Such regulations might be valuable in this context as well. National governments as well as regional institutions and organizations can play a role in facilitating the effective involvement and contributions of migrated health workers [17, 41]. Embassies in the destination countries could be encouraged to work more closely with diaspora associations to facilitate joint ventures and links with the various source countries [41]. Indeed, the development of responsive policy to the Philippine nurse brain drain offers an example of a domestic response [42]. Part of this policy was the establishment of initiatives that facilitate the return and temporary residence of Philippine-born or Philippine-descent nurses at educational institutions, where they would provide training support and workforce development [42]. Another initiative was established by the Southern Africa Network of Nurses and Midwives, a professional association representing nurses and midwives in 14 Southern African countries, which examined the feasibility of integrating members of the Africa-Canada diaspora into initiatives to strengthen the health sector in member countries [41].

#### Strengths and limitations of the study

The results of this study will apply to receiving countries that do not actively recruit and with a migration policy similar to that in Austria and Belgium, and therefore the results are not generalizable to other countries, especially those that have a tradition of active recruitment. Further, given the small number of participants from individual source countries, all of which are diverse in both their socioeconomic and political situations, generalizations about sub-Saharan Africa must be interpreted with caution. Additionally, given that study participation was voluntary, there is a possibility that selection bias may have occurred. However, we managed to compile a relatively large and heterogeneous sample of 27 health workers. Nevertheless, despite the large sample, there was only a small number of nurses (7), although the results do not seem to be closely associated with the specific profession of the respondents. It is important to keep in mind that this study was about intentions, and that there is no evidence that those intentions are predictive of relocation.

#### Further research

To further develop the insights into the patterns of return migration, research into the actual contribution of returnees and possible strategies to maximize the benefits for the source countries are recommended. A systematic review of best practice examples of how to encourage migrants to return or to have an active involvement in the training of health workers or the development of the healthcare system in the source country, as well as additional qualitative studies on those who have returned and their experiences, might be of value.

## Conclusion

The present analysis reveals three main reasons for health workers to remain in Europe, namely family ties, living conditions, and war and conflict in their source country. The reasons to potentially return cited by respondents included reunification with their extended family, a preference to live in their home country, the perception of greater job satisfaction in the source country, and a feeling of responsibility towards the source country. As long as safety and structural stability cannot be guaranteed in source countries, we presume that the number of return migrants will remain rather small. However, many health workers do return for short periods of time and maintain professional links with their source country. Indeed, migrant health workers are a potential resource for the development of source country healthcare systems. National governments and regional organizations should facilitate the engagement of migrant health workers.
